# Negative Xpert HPV Test (Cepheid) With Late Amplification Signals: Is There a Clinical Significance? (the PaPCR Study)

**DOI:** 10.1002/jmv.70915

**Published:** 2026-04-15

**Authors:** Valentin Lemoine, Romain Legros, Sylvain Rosec, Camille Salaun, Adissa Tran‐Minoui, Léa Pilorgé, Sophie Vallet, Anne Le Gourrierec, Pierre Alemany, Pascale Marcorelles, Christopher Payan

**Affiliations:** ^1^ Department of Infectious Agents, Laboratory of Virology Brest University Hospital Brest Bretagne France; ^2^ Present address: Laboratory of Bacteriology and Hospital Hygiene University Hospital of Rennes Rennes France; ^3^ Génétique, Génomique fonctionnelle et Biotechnologies (UMR 1078), Inserm Université de Bretagne Occidentale Brest Bretagne France; ^4^ Clinical Investigation Center (CIC 1412) Brest University Hospital Brest Bretagne France; ^5^ Gynecology unit Brest University Hospital Brest Bretagne France; ^6^ Laboratory of Pathology and Cytology Brest University Hospital Brest Bretagne France

**Keywords:** cancer, genotyping, HPV, human papillomavirus, PCR, sexually transmitted infections

## Abstract

Cervical cancer is the fourth most common cancer among women worldwide, with higher prevalence in low‐resource countries due to limited access to screening and HPV vaccination. Nucleic acid amplification tests (NAATs) such as Xpert HPV (Cepheid), enable rapid detection of high‐risk HPV (HR‐HPV) genotypes. However, the clinical significance of weak and late amplification signals (cycle threshold ≥ 38), interpreted as negative, remains unclear. In this study, a comparison with cytological results aimed to assess their significance. Out of 6846 cervical samples analyzed at Brest University Hospital (Jan. 2022–June 2024), 45 showed late amplification and were reported negative by Xpert. These samples were retested using an in‐house HPV pangenotypic PCR followed by genotyping (LiPA) and were assessed by cytology. Of the 44 available cytology results, 65.9% were normal, 15.9% showed low‐grade squamous intraepithelial lesions (LSIL), and 18.2% were atypical squamous cells of undetermined significance (ASC‐US). No high‐grade squamous intraepithelial lesions (HSIL) were found at initial cytology. Among the 42 samples available for in‐house PCR, 59.5% tested positive for HPV, and 11.9% were confirmed as HR‐HPV genotypes (types 52, 56, 35). Additionally, among patients with available HPV history, 73.3% had a previous positive test. In 91% of these cases, the genotype suspected during the late amplification was concordant with the previous infection. During the 1‐year follow‐up, one patient initially classified as LSIL developed a histologically confirmed HSIL (CIN2) and needed treatment by conization, whereas 3 others were found to have low‐grade CIN1 cell lesions. Overall, for 15 out of 45 patients with weak HPV PCR signal and abnormal cytology (33.3%), clinicians chose to recommend a closer 1‐year follow‐up and not at 5 years as recommended for HPV negative patients. In conclusion, cytological triage and a review of patient history remain crucial to avoid missing at‐risk patients, especially in settings where HPV‐negative women are re‐screened only 5 years later. A multicenter study using Xpert HPV and other automated platforms is recommended to strengthen these findings and further explore potential correlations between viral load and clinical severity.

## 1 Introduction

Cervical cancer is the fourth most common cancer in women worldwide, with 660,000 new cases and 350,000 related deaths in 2022 [1]. Persistent infection with human papillomavirus (HPV), notably high‐risk genotypes of HPV (HR‐HPV), occurs in virtually all cases of cervical cancer [2, 3]. The different genotypes are classified from “carcinogenic to humans” (group 1) to “carcinogenicity not classifiable” (group 3) according to the IARC monograph [3]. Notably, HPV 16, 18, and 45 (group 1) are responsible for 77% of all cervical cancers and should be screened for as a priority by PCR [4]. Viral oncoproteins E6 and E7 drive malignant transformation by inactivating tumor suppressor p53, hindering apoptosis, evading immune surveillance, promoting cell invasion, and disrupting the balance of cellular metabolism [5]. Screening programs that rely on validated HPV nucleic acid tests have improved detection of cervical intraepithelial neoplasia (CIN) and reduced cervical cancer incidence. HR‐HPV tests to be used for screening should reach an optimal balance between clinical sensitivity and specificity for the detection of high‐grade CIN (CIN2 +) and cervical cancer to minimize redundant or excessive follow‐up procedures for HR‐HPV‐positive women without cervical lesions. The clinical thresholds used in validated HPV tests ensure this optimal balance and prevent over‐detection of small amounts of DNA that would not be relevant in cervical cancer screening [6].

In France, screening varies with age: between 25 and 29 years old, cytological examination is recommended every 3 years, following two initial cytology tests performed 1 year apart with normal results, whereas between 30 and 65 years old, nucleic acid amplification tests (NAATs) for the detection of HR‐HPV replace cytological testing. The HR‐HPV test is performed 3 years after the last normal cytological examination and is repeated every 5 years if negative. In case of positive results, a cytological examination is performed. A new HR‐HPV test is performed 1 year later in case of a negative cytological examination [7].

The Xpert HPV test (Cepheid) detects HPV DNA in the E6/E7 genomic region, distinguishing between HPV 16 and 18/45, as well as other HR‐HPV in 3 panels, including HPV31, 33, 35, 52, 58, 51, 59, 39, 56. The test also detects HPV66 and 68, which are not classified in the “carcinogenic” group 1: HPV68 is classified as group 2 A “probably carcinogenic” and HPV66 is classified as group 2B “possibly carcinogenic” by the IARC. This test also includes a Sample Adequacy Check (SAC), confirming the presence of human cell DNA. This real‐time PCR‐based test can provide semi‐quantitative information, with access to cycle threshold (Ct) values, and can be used to approximate the HPV viral load in the sample. Indeed, a high viral load is a marker of the persistence of HPV infections, as well as an indicator of the risk of squamous intraepithelial lesions (SILs) [8, 9]. However, the Xpert technology algorithm sometimes classifies samples as “negative” despite the presence of late amplification curves (Ct above 38) in the raw data. Because of the potential clinical implications of sub‐threshold detections, which may lead to missing oncogenic HPV infections and associated high‐grade squamous intraepithelial lesions (HSIL), postponing follow‐up testing—and thereby delaying appropriate patient management—poses a substantial challenge for clinical management.

Some cases of invasive cervical cancer are initially HPV‐negative or show low‐level HPV detection in PCR testing [10, 11, 12, 13]. Various factors are likely to explain this situation: very early infection, resolving infection, transient contamination of the patient, poor sample quality (*e.g*., high DNA fragmentation, sample not representing the lesion, necrosis), and clinical and/or pathological issues (*e.g*., error in designating the primary lesional site, metastasis to cervix from other cancer primary site). In addition, sensitivity is known to differ for each HPV genotype (*e.g*., HPV 33) [14].

Several studies have evaluated the clinical significance of low HPV viral loads in cervico‐uterine swabs using different PCR systems, but to our knowledge, no studies have been conducted on Xpert.

A first study using Anyplex II HPV28 Detection Assay (Seegene, Seoul, Republic of Korea) was conducted by Park et al. In this study, among positive samples after 40 cycles of PCR (with low viral loads), most cytology results were negative, except for 7 cases with atypical squamous cells of undetermined significance (ASC‐US) and 2 cases with atypical glandular cells. The authors considered that, since cytology is negative in most cases when the viral load is low, there is no need for further investigations [15].

A second study using the Alinity m HR HPV Assay (Abbott, Chicago, USA) was conducted by Node et al. In this study, 1.7% of tested samples reported as negative by the Alinity algorithm showed a late amplification signal below the specific clinical threshold of the assay, and among them, no high‐grade lesions were confirmed by histology. However, during the 1‐year follow‐up, 2 patients were confirmed with CIN2 and CIN3 lesions. The authors recommended close follow‐up for these cases [13].

Therefore, the aim of this study was (1) to determine the significance of these late amplification curves with regard to cytology results to assess the presence or absence of HSIL and (2) to evaluate the significance of Xpert technology with late amplification curves using an in‐house real‐time HPV qPCR and genotyping test as described previously [16].

## 2 Materials and Methods

### 2.1 Samples Selection

**Figure 1:**
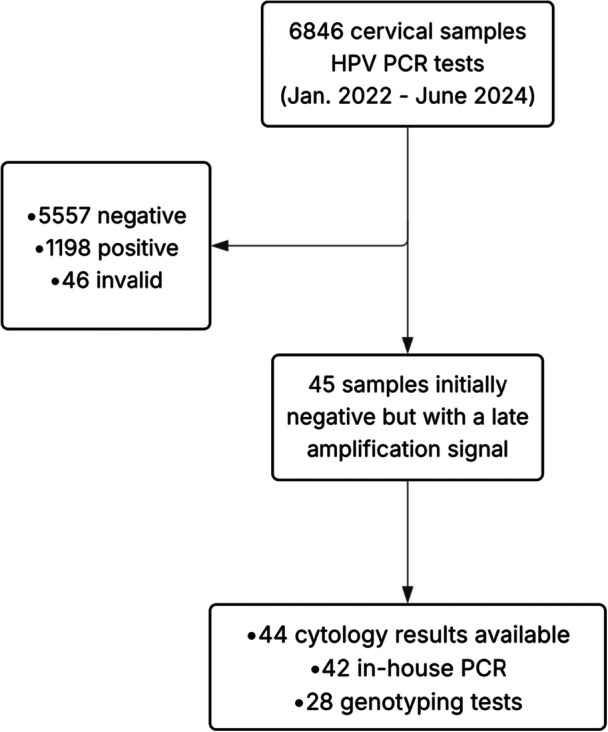
Study workflow for cervical samples selection.

Adult women (≥ 18 years) with cervical samples performed for HPV screening from January 2022 to June 2024 in the Gynecology unit, Brest University Hospital, were eligible. Cervical samples were collected using a cytobrush and transferred into ThinPrep PreservCyt medium (Hologic). Samples were stored at room temperature (< 4 days) until analysis. Aliquots were kept in cryotubes at −80°C for long‐term storage. Only cervical samples showing late amplification signals were included in this study (Figure 1).

### 2.2 Xpert HPV Testing

HPV testing was performed using the Xpert HPV test (GeneXpert, Cepheid, Berkeley, USA), with the Infinity‐48s automated from Cepheid, according to the manufacturer's instructions. Each cartridge contains the extraction, amplification, and detection processes, with lyophilized beads containing all PCR reagents: Taq polymerase, dNTPs, buffer, primers, and HPV probes, and an internal cell control (SAC) detecting the presence of a single‐copy human gene. The result is considered valid only if the cell control is positive (Ct < 37). Amplification of the E6 and E7 genes of 14 HR‐HPV types is performed simultaneously with five fluorescence channels, grouping HR‐HPVs in the order indicated in Table 1.

**Table 1:** Xpert HPV fluorescence channels, with the different HPV genotypes detected for each channel.

Channel	Xpert channel denomination	HPV genotypes detected					Clinical threshold (Ct)
P1	HPV 16	16					≤ 40
P2	HPV 18_45	18	45				≤ 40
P3	P3	31	33	35	52	58	≤ 38
P4	P4	51	59				≤ 38
P5	P5	39	56	66	68		≤ 38

Depending on the HPV channels P1 to P5, the Ct limit for a positive result is different. For HPV 16 (P1), 18/45 (P2), the Ct must be below 40, whereas for other HR‐HPVs in P3, P4, and P5, the Ct must be below 38. However, Ct value is available with raw data results when a late amplification signal is observed, even if negative results are given by the algorithm of the Xpert. As PCR is not quantitative, the manufacturer does not recommend using Ct values [17]. We estimated the viral load by calculating the ΔCt between the HPV target and the SAC (see *2.7. Viral Load Estimation*). For samples returning a “negative” Xpert result that nonetheless displayed amplification curves indicative of a low viral load, the samples were sent to the Pathology and Cytology laboratory (Brest University Hospital) for cytological examination.

### 2.3 Cervical Cytology

Cytological analysis of the samples was performed with the Genius Digital Diagnostics System (Hologic) using the Bethesda 2014 grading criteria by the Pathology and Cytology laboratory at Brest University Hospital. Abnormal results included atypical squamous cells of undetermined significance (ASC‐US), atypical squamous cells that cannot exclude high‐grade squamous intraepithelial lesion (ASC‐H), low‐grade intraepithelial lesions (LSIL), and high‐grade intraepithelial lesions (HSIL). p16 staining (CINtec p16 Histology, Roche Diagnostics) was performed on biopsy samples for certain patients who required colposcopy during follow‐up.

### 2.4 Clinical and Biological Data

Clinical and biological information on patients (age, HPV test results, cytology and colposcopy results where applicable, medical history, and follow‐up where available) was obtained retrospectively by consulting the laboratory information management system (Glims, Clinisys) and the clinical database (M‐EVA, Maincare).

### 2.5 In‐House Pangenotypic Quantitative PCR

In‐house quantitative PCR was adapted from a protocol published in 2007 [16]. Briefly, extraction of samples stored at −80°C was performed on EMAG (bioMérieux, Marcy‐l’Étoile, France) using 1 mL of sample for an eluate volume of 50 µL. HPV DNA amplification and quantification were performed on LightCycler 480 (Roche Diagnostics, Meylan, France). The protocol uses SYBR Green, as well as sense and antisense primers covering more than 50 HPV genotypes in the L1 region, and combines an HPV 16 probe. A GAPDH gene cell control is amplified in parallel, as well as a marine phage MCP gene as an internal control.

### 2.6 HPV Genotyping

A nested PCR was performed using SPF10 primers on the in‐house qPCR products. Genotyping of these amplicons was performed after reverse hybridization with HPV probes on strips, using the LiPA (Line Probe Assay, Fujirebio) method with the Tendigo automated system (Fujirebio).

### 2.7 Viral Load Estimation

As a relative estimate of viral load, we calculated ΔCt values for available samples. For Xpert HPV results, ΔCt was defined as: ΔCt_GX_ = (Ct_HPV_) − (Ct_SAC_). For the in‐house pangenotypic PCR, ΔCt was defined as: ΔCt_conv_ = (Ct_HPV_) − (Ct_GAPDH_). These ΔCt values were calculated only when both target and reference Ct were available and < 40 and were used to compare relative signal strengths between assays; they do not represent absolute viral copy numbers. The ΔCt values shown are inversely proportional to the HPV viral load.

### 2.8 Statistical Analysis

Descriptive statistics were used to report the median with the 25th–75th quartiles in a univariate analysis. Concordance between the Xpert assay and the reference genotyping method was evaluated for samples testing positive by in‐house PCR (Ct < 40). Samples where genotyping was not performed were excluded from the analysis. A result was defined as concordant when the specific genotype identified by LiPA matched the corresponding fluorescent channel (P1–P5) of the Xpert test, as detailed in Table 1. Conversely, samples testing positive by in‐house PCR but returning a negative, non‐typable (NT), or mismatched genotype by LiPA were classified as discordant. To compare ΔCt values between each cytology group, the nonparametric Kruskal‐Wallis test was used, as appropriate. *p‐values* < 0.05 were considered significant. GraphPad Prism (version 8.0.1) was used for the analysis and generation of graphs.

## 3 Results

### 3.1 Patients and Xpert HPV Tests

From January 2022 to June 2024, a total of 6846 HPV PCR tests were performed. The mean age of patients was 44.8 years (SD = 12.2). Among these samples, 5557 (81.2%) were negative, 1198 (17.5%) were positive, and 46 (0.67%) were rendered invalid due to late cell control amplification (Ct_SAC_). Another 45 (0.66%) samples were initially reported as negative through the Xpert algorithm, although a Ct and endpoint signal were indicated in the raw data. Of the 1198 positive samples, 24.3% were infected with HPV16 (alone or in co‐infection), 12.9% were infected with HPV18 (alone or in co‐infection), 76.1% were infected with another HR‐HPV (alone or in co‐infection), and 13.3% were co‐infections.

### 3.2 Overall Cervical Samples With Negative Results and Late Amplification Curves

Cytological analysis was carried out on 44 of the 45 samples. There were 8 ASC‐US (18.2%), 7 LSIL (15.9%), and 29 with normal cytology (65.9%). The raw results regarding cytology results are presented in Tables 2, 3, 4.

**Table 2:** Summary table of Xpert and in‐house PCR data, as well as genotyping data if performed, and results of colposcopy, for patients with ASC‐US at cytology.

Patient number	Age	Xpert results				In‐house PCR followed by genotyping (LiPA)					Concordance	Colposcopy/Histological examination
		HPV channel	Ct_HPV_	Ct_SAC_	ΔCt_GX_	Ct_conv_	Ct_GAPDH_	ΔCt_conv_	Genotyping	IARC classification		
6	34	P5	38.3	35.1	3.2	31.8	29.9	1.9	66, 62	2B, 3	Yes	No follow‐up available
18	54	P4	38.4	26.1	12.3	ND	ND	ND	ND		ND	Normal
21	37	P3	38.8	27.1	11.7	38.8	26.7	12.1	**52**	**1**	Yes	Low‐grade CIN1 cell lesions confirmed after 1 year follow‐up
27	27	P5	39.8	28.5	11.3	36.6	25.9	10.7	NT		No	No follow‐up available
32	30	P5	39.0	37.4	1.6	32.3	28.4	3.9	81	3	No	No follow‐up available
34	34	P4	39.9	26.8	13.1	37.3	26.0	11.3	81	3	No	Low‐grade CIN1 cell lesions after 1 year follow‐up
41	42	P5	38.5	29.7	8.8	40.0	26.9	ND	ND		ND	Normal
45	45	P3	39.2	25.1	14.1	35.1	25.1	10.0	**52**, 44	**1**, INC	Yes	Normal

*Note:* ΔCt_GX_ = Ct_HPV_ – Ct_SAC_; ΔCt_conv_ = Ct_conv_ – Ct_GAPDH_; P3 corresponds to genotypes 31, 33, 35, 52, and 58; P4 corresponds to genotypes 51 and 59; P5 corresponds to genotypes 39, 56, 66, and 68.

Abbreviations: ND, not determined; NT, non‐typable with the LiPA method; SAC, sample adequacy control (i.e., DNA cell control).

**Table 3:** Summary table of Xpert and in‐house PCR data, as well as genotyping data if performed, and results of colposcopy, for patients with LSIL at cytology.

Patient number	Age	Xpert results				In‐house PCR followed by genotyping (LiPA)					Concordance	Colposcopy/Histological examination
		HPV channel	Ct_HPV_	Ct_SAC_	ΔCt_GX_	Ct_conv_	Ct_GAPDH_	ΔCt_conv_	Genotyping	IARC classification		
8	34	P5	40.0	23.8	ND	31.7	24.6	7.1	66, 67	2B, 2B	Yes	Normal
16	43	P5	37.4	30.1	7.3	25.8	25.3	0.5	**56**, 81	**1**, 3	Yes	Normal but low‐grade CIN1 cell lesions after 1 year of follow‐up
19	46	P3	38.5	25.6	12.9	ND	ND	ND	ND		ND	High‐grade CIN2 squamous cell lesions and conization after 1 year of follow‐up
24	63	HPV16	38.0	23.3	14.7	27.5	23.2	4.3	89	3	No	No follow‐up available
37	32	P5	38.8	25.1	13.7	38.2	24.8	ND	Negative		No	Normal
39	34	P4	38.5	28.5	10	40.0	26.9	ND	ND		ND	Normal
44	42	P3	39.0	31.8	7.2	33.9	26.5	7.4	62	3	No	Normal

*Note:* ΔCt_GX_ = Ct_HPV_ – Ct_SAC_; ΔCt_conv_ = Ct_conv_ – Ct_GAPDH_; P3 corresponds to genotypes 31, 33, 35, 52, and 58; P4 corresponds to genotypes 51 and 59; P5 corresponds to genotypes 39, 56, 66, and 68.

Abbreviations: ND, not determined; Negative, no control band for genotyping by LiPA; NT, non‐typable with the LiPA method; SAC, sample adequacy control (i.e., DNA cell control).

**Table 4:** Summary table of Xpert and in‐house PCR data, as well as genotyping data if performed, for patients with normal cytology results.

Patient number	Age	Xpert results				In‐house PCR followed by genotyping (LiPA)					Concordance
		HPV channel	Ct_HPV_	Ct_SAC_	ΔCt_GX_	Ct_conv_	Ct_GAPDH_	ΔCt_conv_	Genotyping	IARC classification	
1	53	P5	40.0	27.0	ND	40.0	25.8	ND	ND		ND
2	39	P5	39.4	25.8	13.6	40.0	25.2	ND	ND		ND
3	30	P5	40.0	31.6	ND	40.0	29.1	ND	ND		ND
4	47	P5	40.0	35.6	ND	37.4	25.9	11.5	66, 62, 81	2B, 3, 3	Yes
5	45	P5	40.0	28.5	ND	36.9	25.7	11.2	81	3	No
7	31	P3	38.7	27.5	11.2	40.0	26.7	ND	ND		ND
9	31	P5	39.0	25.6	13.4	34.8	25.0	9.8	62, 83	3, 3	No
10	32	P5	40.0	30.8	ND	38.7	25.8	ND	Negative		No
11	31	P3	38.2	29.5	8.7	34.8	26.7	8.1	82, 67	2B, 2B	No
12	62	P4	38.6	28.9	9.7	40.0	24.8	ND	ND		ND
13	30	P5	38.2	30.3	7.9	28.6	27.7	0.9	**35**, 67, 81	**1**, 2B, 3	No
14	53	P5	40.0	29.8	ND	22.2	25.1	‐2.9	53, 66, 89	2B, 2B, 3	Yes
15	27	P4	38.8	31.1	7.7	35.8	29.3	ND	Negative		No
17	51	P4	38.3	29.6	8.7	30.7	26.3	4.4	06, 53, 70	3, 2B, 2B	No
20	34	HPV16	40.0	27.4	ND	40.0	25.8	ND	ND		ND
23	41	P3	38.4	25.6	12.8	37.8	25.0	12.8	89	3	No
25	35	P4	38.8	32.8	6.0	33.1	29.5	3.6	89	3	No
26	64	P4	38.3	29.2	9.1	40.0	26.4	ND	ND		ND
28	28	P5	39.4	25.0	14.4	40.0	23.8	ND	ND		ND
29	69	P3	39.1	28.1	11.0	36.5	26.8	ND	Negative		No
30	44	P5	39.0	29.2	9.8	30.9	27.2	3.7	66	2B	Yes
31	37	P5	38.9	35.0	3.9	ND	ND	ND	ND		ND
33	62	P5	39.6	32.8	6.8	32.9	27.3	5.5	66	2B	Yes
35	70	P5	40.0	27.9	ND	38.3	25.0	13.3	NT		No
36	33	P4	38.7	25.4	13.3	40.0	26.1	ND	ND		ND
38	39	P5 + P3	39.9	25.5	14.4	39.1	24.6	14.5	**52**	**1**	Yes
40	29	P4	39.0	26.3	12.7	38.2	25.1	13.1	ND		ND
42	63	P3	39.2	25.2	14.0	40.0	24.3	ND	ND		ND
43	30	P5	39.5	33.9	5.6	37.1	31.4	5.7	66	2B	Yes

*Note:* P3 corresponds to genotypes 31, 33, 35, 52, and 58; P4 corresponds to genotypes 51 and 59; P5 corresponds to genotypes 39, 56, 66, and 68.

Abbreviations: ND, not determined; Negative, no control band for genotyping by LiPA; NT, non‐typable with the LiPA method; SAC, Sample Adequacy Control (i.e., DNA cell control).

Of the 44 samples whose cytology was analyzed, a Kruskal‐Wallis test was performed between each group (normal cytology, ASC‐US, LSIL) on the ΔCt_GX_ (*n* = 35) and on the ΔCt_conv_ (*n* = 25). The comparison of viral loads was performed only on samples with a detectable signal in both tests (Ct < 40 and excluding negative samples after genotyping in the in‐house PCR group). Individual ΔCt values are provided in Tables 2, 3, 4.

**Figure 2:**
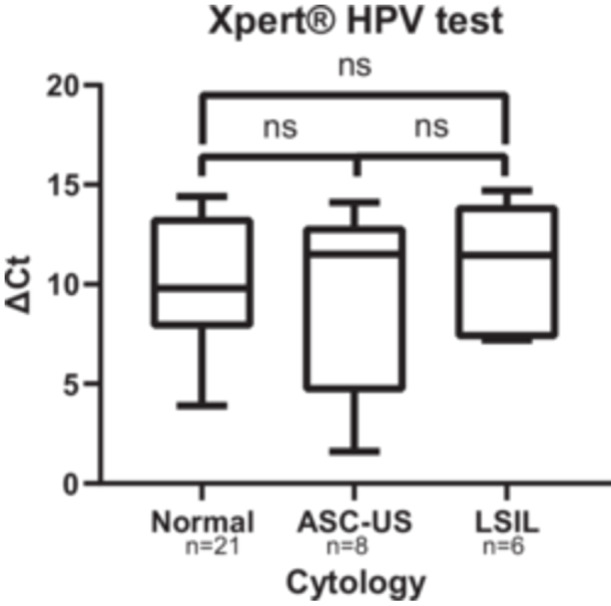
Analysis of the ΔCT detected using Xpert HPV test (ΔCt_GX_) in the different cytology groups; ns: not significant (*p* value > 0.05).

For the analysis of Xpert data, we excluded samples with no amplification (Ct ≥ 40). Consequently, 35 samples were analyzed, including 21 with normal cytology, 8 ASC‐US, and 6 LSIL. For normal cytology, the median ΔCt_GX_ was 9.80 [IQR:7.80–13.35]. For the ASC‐US group, the median was 11.5 [4.60–12.90]. Finally, for the LSIL group, the median was 11.45 [7.20–13.95]. No significant difference was observed between the groups regarding ΔCt_GX_ (*p* = 0.82) (Figure 2).

**Figure 3:**
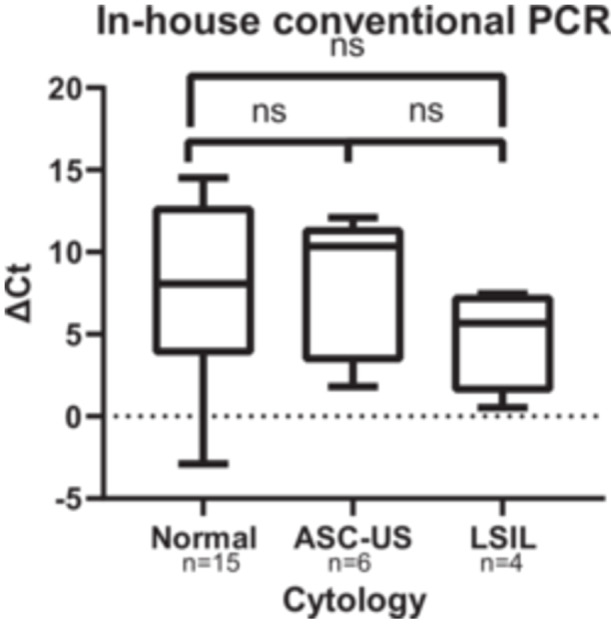
Analysis of the ΔCT detected using in‐house conventional PCR (ΔCt_conv_) in the different cytology groups; ns, not significant (*p* value > 0.05).

For the analysis of in‐house conventional PCR data, we excluded samples with no amplification (Ct ≥ 40) as well as those with positive amplification but negative genotyping results, to ensure signal specificity. Consequently, 25 samples were analyzed, including 15 normal cytology, 6 ASC‐US, and 4 LSIL. For the normal cytology group, the median ΔCt_conv_ was 8.07 [3.75–12.79]. For the ASC‐US group, the median was 10.34 [3.32–11.50]. Finally, for the LSIL group, the median was 5.68 [1.47–7.36]. Although the viral load appears to be higher in the LSIL group (lower ΔCt_conv_) compared to the normal cytology group, no statistically significant difference was observed (*p* = 0.45) (Figure 3).

For patients 18, 19, and 31, we were unable to perform in‐house PCR or genotyping due to insufficient sample quantity stored in the laboratory. Patient 31 had normal cytology. For patient 18, ASC‐US was found on cytology, and follow‐up a few months later found no abnormalities. For patient 19, LSIL was found on cytology. A few months later, this patient progressed to HSIL/CIN2 (*see 3.6 Samples With LSIL*).

### 3.3 Previous Results of the Xpert HPV Test for Included Patients

Previous Xpert HPV test results were available for only 15 out of 45 patients (33.3%). Of these, four patients had previously tested negative, while 11 had previously tested positive, 10 of whom had potentially identical genotypes (with at least one identical P3, P4, or P5 fluorescence channel in comparison with the Xpert results) (Table 5).

**Table 5:** Previous results of the Xpert HPV test for included patients.

Previous Xpert HPV test results	*n* = 15
Negative	4 (26.7%)
Positive	11 (73.3%)
Possibly identical genotype (at least one identical fluorescence channel)	10
Other genotype	1

### 3.4 Xpert HPV Test for Follow‐Up of Included Patients

Xpert HPV PCR screening was repeated as part of the follow‐up process for only 7 patients (15.6%) in our hospital, after a follow‐up period of at least 1 year. This 1‐year follow‐up was proposed by the clinicians as a precautionary measure for 15 women with abnormal cytology (8 ASC‐US and 7 LSIL) among the 45 showing a weak PCR signal in our study (33.3%). The test was negative for 4 patients, meaning they could be followed up again in 5 years. However, 3 patients tested positive for HPV DNA. 2 women had a genotype possibly identical to the previous one (same fluorescence channel), and 1 woman had a modified genotype (P3 to P5) (Table 6).

**Table 6:** Xpert HPV test for follow‐up of included patients.

Xpert HPV test for follow‐up(after at least 1 year of follow‐up)	*n* = 7
Negative	4 (57.1%)
Positive	3 (42.9%)
Possibly identical genotype (at least one identical fluorescence channel)	2
Other genotype	1

### 3.5 Samples With ASC‐US

Eight women with a median age of 36 [33–43] had ASC‐US (Table 2). Out of the 7 samples tested with the in‐house PCR, 6 were confirmed positive, showing a 50% concordance rate between the Xpert probes and LiPA genotypes. Three of these patients had a known history of positive HPV testing with the corresponding genotype.

Genotyping identified HPV52 in two cases (100% concordance with the Xpert P3 channel), while the other four cases involved group 2B (HPV66) or group 3 (HPV62, 81) genotypes.

Patient 21 had HPV52 and was under immunosuppressive treatment for a renal transplant. Prior testing 1 year before had shown persistent HR‐HPV (P3 channel) and low‐grade lesions on cytology. During follow‐up at 1 year after testing with a weak PCR signal in P3, cytology testing showed LSIL, confirmed by colposcopy, which required close annual monitoring. Follow‐up 2 years later showed the persistence of HPV but with a different genotype according to the Xpert technique. In fact, this patient had repeatedly tested positive for the P3 channel genotype, and 2 years later, she tested positive for the P5 channel genotype. The last biopsy performed showed condyloma without significant overexpression of the p16 marker.

Patient 45 also carried HPV52. HR‐HPV was found in the smear 5 years before, with persistent P3 and no suspicious lesion on colposcopy. No follow‐up was available for this patient.

For patient 34, no previous HPV testing was available, but during follow‐up at one and 2 years, HPV was found positive with the same genotype (*i.e*., fluorescence channel P4) and cervical biopsy revealed low‐grade lesions and condyloma.

### 3.6 Samples With LSIL

Seven women with a median age of 42 [34–44] had LSIL (Table 3). Six were tested by in‐house PCR, with four confirmed positive, yielding a concordance rate of 40%. Among the four patients with prior HPV testing, two had previous positive results with the same genotype. Five patients underwent follow‐up: three cleared the virus after 1 year, while two (patients 16 and 19) remained positive.

Patient 16 (HPV56) was already known to have HR‐HPV (other than HPV16 or 18) and had a cytology classified as ASC‐H. Colposcopy was also satisfactory, with no observed lesions. However, follow‐up at 1 year showed persistence of the same HPV genotype group (P5) and revealed grade 1 atypical changes only in the endocervix at cervical biopsy, with negative p16 staining.

Patient 19 had undergone a first conization 6 months prior with a known HPV P3 positive test (Ct = 35) and CIN2 lesions. At the 6‐month control HPV test, she presented a late amplification signal on channel P3 (Ct = 38.5), which was also reported as negative by the Xpert algorithm. In‐house PCR could not be performed due to insufficient sample volume. Initial cytology revealed low‐grade lesions LSIL. During the 1‐year follow‐up, this patient showed persistence of the same HPV P3 (Ct = 32.7), which was reported positive and developed a histologically confirmed HSIL (CIN2, with positive p16 staining), which required conization.

For the other 5 patients, the colposcopies performed did not show any particular abnormalities.

### 3.7 Samples With Normal Cytology

Among the 45 women with a weak HR‐HPV PCR signal, 29 (64.4%) had normal cytology, with a median age of 39 [31–53] (Table 4). Of the 28 samples tested by in‐house PCR, 18 were confirmed positive, and 13 genotypes were successfully identified, yielding an overall concordance rate of 35.3%.

Among these 29 patients, 8 had previously undergone an HPV Xpert test at our hospital. 2 patients (23 and 31) had received a negative test result, and 6 others (patients 10, 11, 13, 15, 17, and 25) had a history of positive PCR tests. Among the 6, the same genotype group was found in 5 cases. Only patient 17 had a genotype change from P5 to P4. Another patient (no. 15) had a coinfection with HR‐HPV group P4 (Ct = 33.8) and P5 (Ct = 30.6) and had conization 2 years before and was found with a weak signal in P4 (Ct = 38.8) reported negative by the Xpert algorithm. In one patient (no. 11), with a weak PCR signal in P3 (Ct = 38.2), HR‐HPV was detected 1 year before, with coinfection P3 (Ct =33.1) and P5 (Ct = 38.0), and cytology classified as ASC‐US. However, a sample taken 1 year later found no HR‐HPV. For another patient (no. 25), with a weak PCR signal in P4 (Ct = 38.8), HR‐HPV was detected 1 year before in the same genogroup P4 (Ct = 31.1) with cytology classified as LSIL. No follow‐up at 1 year was reported for this patient.

For two samples, HR‐HPV was confirmed by LiPA genotyping, with HPV35 and HPV52. Of these two samples, patient 38 was positive for P3 (and P5), which matched the HPV‐52, while patient 13 was positive for P5, but genotyping identified HPV35, a type that should have been detected on the P3 channel, showing some discordance.

## 4 Discussion

This study aimed to explore the clinical significance of late HPV amplification interpreted as “negative” results by the Xpert system. Among 6846 cervical smears screened retrospectively, we found 45 samples meeting the inclusion criteria. This is a rare event, representing only 0.66% of the whole population screened during a 2.5‐year period in this retrospective study. This proportion is lower than that reported in previous studies by Node (1.7%) [13] and Park (5.99%) [15]. In this cohort, no cases of HSIL were found among these cervical samples, with late amplification interpreted as “negative” by the Xpert algorithm at the time of initial testing. However, 15 cases with abnormal cytology (33.3%, 8 ASC‐US and 7 LSIL), which led clinicians to propose a closer surveillance than the routine 5‐year screening interval as recommended for women with negative HPV testing.

Our findings extend those reported in two studies by Node et al. [13] and Park et al. [15] using the Alinity m (Abbott Molecular) and Anyplex (Seegene Inc) systems, respectively. However, in our study, we further investigated these samples using an in‐house PCR and LiPA genotyping in order to characterize the HPV genotypes potentially associated with these late amplification curves.

Out of the 42 samples tested by in‐house PCR, only 5 HR‐HPV (IARC group 1) were detected: 2 cases with normal cytology, 2 with ASC‐US, and 1 with LSIL, *i.e*., 11.9% classified as negative according to the clinical threshold of the Xpert algorithm. After genotyping these 5 HR‐HPV types, we obtained 3 HPV52, 1 HPV56, and 1 HPV35, which had initially tested negative using the Xpert HPV test. Our results show good overall agreement between genotyping performed using the LiPA HPV test and the pool of genotypes in the Xpert HPV test (*i.e*, 4 out of 5). Only 1 in 5 samples showed HPV35 amplified in the wrong pool in the Xpert HPV test (P5 instead of P3 as expected). Interestingly, HPV52 and HPV35 probes are part of the P3 pool, which showed a lower Ct signal level of positivity (Ct < 38); if over 38, the results of the Xpert algorithm are negative. Thus, the probability of observing a late amplification signal is higher with P3 probes than others (*i.e*., HPV16, 18, or 45 or P5) with a Ct cut‐off at 40. However, while techniques like LiPA are very sensitive due to the small size of the amplicons and are useful for genotyping, they have low specificity for cervical cancer screening, as demonstrated by the VALGENT framework, because the detection of minute amounts of HPV DNA is often not biologically relevant [18, 19]. It is important to remember that the Xpert HPV test has been clinically validated for screening purposes within the VALGENT framework, demonstrating sufficient clinical accuracy for CIN2+ lesions [20, 21].

An important consideration is that the clinical positivity thresholds of the Xpert assay are not identical across target channels or HPV genotype pools. Furthermore, the detection of HR‐HPV depends on the number of copies present in the sample, which may be affected by sampling conditions, patient factors, stage of infection, or interfering parameters. These interferences include the presence of whole blood (≥ 0.25% v/v) and leukocytes (≥ 1 ×105 cells/mL), vaginal candidiasis caused by *Candida albicans* (≥ 1 ×10⁸ cells/mL), and vaginal creams or gels [17]. Our in‐house pangenotypic PCR, while capable of detecting a broad range of genotypes, may differ in analytical and clinical thresholds (highly sensitive, about 20 HPV DNA copies). Differences in genomic targets must also be considered. Our in‐house PCR targets the L1 capsid region, whereas the Xpert test targets the E6 and E7 oncogenes. This difference is critical in the event of viral integration, a key step in cervical carcinogenesis. During integration of the viral genome into the host DNA, the L1 region is frequently disrupted or deleted “L1 drop‐out”, rendering L1‐based tests potentially false‐negative in the presence of precancerous or cancerous lesions [22]. Conversely, E6 and E7 genes are invariably retained and overexpressed to maintain the oncogenic process, ensuring their detection by Xpert even in cases of complete integration. This distinction could explain why some samples exhibit a late Xpert signal (E6/E7 detected) while conventional PCR (L1) remains negative, potentially reflecting early integration rather than simply a low viral load. These methodological differences should be considered when interpreting discordant results.

A key strength of this study is the longitudinal follow‐up of patients presenting these late amplification signals. Similar to observations by Node et al. [13] on the Alinity m system, we found that the majority of patients for whom history was available (15/45, 33.3%) had a history of positive HPV tests (11/15, 73.3%). Most importantly, in 90.9% of these positive cases (10/11), the genotype (or fluorescent channel group) suspected during late amplification was concordant with the previous infection. This observation reinforces the hypothesis that these “negative with late curve” results often reflect persistent infection with fluctuating viral load rather than background noise. While Node et al. [13] reported 58% of patients with identical genotypes in their history, our data corroborates this trend with the Xpert test. Regarding follow‐up, Xpert HPV PCR screening was repeated for only 7 patients (15.6%). Among them, 3 tested positive for HPV DNA, with 2 women showing a genotype possibly identical to the initial late signal. This persistence, even at low viral loads, warrants specific attention.

The clinical significance of these late signals is particularly highlighted by the case of patient 19. Despite a negative Xpert result, the concurrent LSIL cytology prompted a follow‐up that eventually revealed a CIN2 lesion requiring surgery. While this isolated observation does not question the clinical validation of HPV assays for primary cervical cancer screening, it highlights the importance of careful clinical interpretation in specific contexts, such as recently treated patients and the follow‐up of patients with persistent HPV.

The correlation between viral load (*i.e*., estimated by Ct values) and the cytology results is still debated, and research studies are ongoing on this subject. Viral load appears to be a marker of disease progression in several studies [23, 24, 25, 26]. Indeed, persistent infections with high viral loads were more likely to cause cervical lesions, while persistent infections with low viral loads were less likely to progress [23]. HPV16, 18, 52, and 58 viral loads are risk factors for the development of cervical lesions. In particular, HPV16 and HPV58 have respectively demonstrated a non‐linear association with the emergence of CIN1+ and CIN2+ cervical lesions, indicating that HPV viral load can serve as a stratification marker to recognize an increased risk of cervical lesions, thereby improving risk stratification in cervical cancer screening [24]. Another study [25] demonstrated that viral load can be used as a triage indicator for HPV52‐positive patients. Wang et al. reported that viral loads of HPV16, 31, 35, 52, 58, 39, and 56 were lower in women with normal cytology compared to those with disease progression, but viral loads were not appreciable for HPV33, 18, 45, 59, 68, 53, 66, and 51 [27]. However, another study has shown that a single viral load measurement taken at an indeterminate point in the natural course of HPV infection cannot reliably predict the risk of developing cervical neoplasia. Therefore, a single HPV viral load measurement cannot be considered a clinically useful biomarker [28]. Age and viral integration also impact the viral load [29].

This study has some limitations. First, the sample size of the ‘late amplification’ group is relatively small (*n* = 45), which limits the statistical power to detect significant differences in viral loads between cytology groups. Consequently, the absence of statistical significance reported in our results does not definitively rule out a correlation between viral load and lesion severity in this specific population. It is a retrospective study covering a period of 30 months with data from a single hospital. Vaccination status could not be retrieved from medical records for most patients included in this study. Based on the birth year distribution of our subcohort of 45 patients (mean age 41.5 years, SD 12.4), approximately 47% of patients were born before 1984 and were therefore ineligible for the HPV vaccination program introduced in France in 2007, which targeted girls aged 14 years with catch‐up vaccination up to 23 years of age [30]. Furthermore, HPV vaccination coverage in France has historically been very low, not exceeding 10% for the complete schedule among the eligible catch‐up age group at program launch [31], making it unlikely that a substantial proportion of the remaining patients had been effectively vaccinated. Future prospective multicenter studies should systematically record vaccination status to further characterize its potential influence on persistent low‐level HPV detection. In addition, the follow‐up period is short, and some patients were lost to follow‐up. A multicenter trial with longitudinal follow‐up of patients with confirmation of positivity by in‐house PCR, followed by genotyping, would allow us to see if there are genotypes that are more likely to be amplified late with the Xpert HPV test associated with clinical significance.

## 5 Conclusion

In conclusion, samples with a late amplification signal and reported as negative by the automated Xpert system are found in rare cases (less than 1% of screened patients). Given the low rate of oncogenic HPV genotypes among these cases, our results do not support the routine use of in‐house PCR followed by HPV genotyping for each sample reported as negative by the Xpert algorithm, which would be costly and unnecessary for patient management. However, cytology may be recommended in these cases to help clarify the interpretation of these late amplification samples, to ensure patients are not lost to follow‐up (*i.e*., more than 33% of these cases had abnormal cytology). Reviewing prior HPV history is critical, as late‐amplification samples often represent persistent infections rather than sporadic background signals. Thus, 3 patients from this cohort underwent conization. In the context of the French screening program, where HPV‐negative women are only re‐screened after 5 years, relying solely on the automated cut‐off could be hazardous. The progression to a histologically confirmed HSIL (CIN2) observed in one patient in this study underscores the clinical risk of delaying follow‐up. Therefore, we advocate for a cautious approach involving cytological review to prevent missing at‐risk patients. A multicenter study involving Xpert and other automated platforms is warranted to validate these findings and refine management algorithms.

## Author Contributions

**Valentin Lemoine:** writing – original draft, formal analysis, investigation, conceptualization, data curation. **Romain Legros:** writing – original draft, testing validation. **Sylvain Rosec:** data curation, writing – review and editing. **Camille Salaun:** data curation. **Adissa Tran‐Minoui:** visualization and testing validation. **Léa Pilorgé:** visualization and testing validation. **Sophie Vallet:** visualization and testing validation. **Anne Le Gourrierec:** inclusion of patients and visualization. **Pierre Alemany:** cytology and visualization, **Pascale Marcorelles:** cytology and visualization. **Christopher Payan:** writing – review and editing, supervision, project administration; methodology, conceptualization, investigation.

## Funding

The authors have nothing to report.

## Disclosure

Part of this manuscript's results have been previously presented at the BiomedJ congress, May 14‐15, 2025, Paris, France.

## Ethics Statement

This study is registered under the number NCT06919627 and was approved by the Brest University Hospital Ethics Committee on March 6, 2025 (no. 29BRC25.0053).

## Consent

All study participants provided informed consent to participate, in line with French ethical guidelines.

## Conflicts of Interest

The authors declare no conflicts of interest.

## Data Availability

The data that support the findings of this study are available from the corresponding author upon reasonable request.
